# Characterization of COVID‐19‐Associated Candidemia Among Burn Patients

**DOI:** 10.1002/jcla.70031

**Published:** 2025-04-08

**Authors:** Maryam Salimi, Javad Javidnia, Azam Moslemi, Mahdi Abastabar, Mohammad Reza Mobayen, Golnar Rahimzadeh, Nahid Mirzaei Tirabadi, Seyedehzahra Nouranibaladezaei, Hassan Asghari, Behnam Sobouti, Mostafa Dahmardehei, Seyedmojtaba Seyedmousavi, Tahereh Shokohi

**Affiliations:** ^1^ Student Research Committee School of Medicine, Mazandaran University of Medical Sciences Sari Iran; ^2^ Invasive Fungi Research Center Communicable Diseases Institute, Mazandaran University of Medical Sciences Sari Iran; ^3^ Department of Medical Mycology School of Medicine, Mazandaran University of Medical Sciences Sari Iran; ^4^ Burn and Regenerative Medicine Research Center Guilan University of Medical Science Rasht Iran; ^5^ Pediatric Infectious Diseases Research Center Communicable Diseases Institute Mazandaran University of Medical Sciences Sari Iran; ^6^ Department of Infectious Disease and Tropical Medicine Shahid Motahari Burns Hospital, Iran University of Medical Sciences Tehran Iran; ^7^ Burn Center Zare Hospital, Mazandaran University of Medical Sciences Sari IR Iran; ^8^ Infectious Disease Research Center Ali‐Asghar Children Hospital, Iran University of Medical Sciences Tehran Iran; ^9^ Department of Plastic and Reconstructive Surgery Burn Research Center, Iran University of Medical Sciences Tehran Iran; ^10^ Microbiology Service Department of Laboratory Medicine, Clinical Center, National Institutes of Health Bethesda Maryland USA

**Keywords:** burn patients, Candidemia, COVID‐19, fungal infection, wound colonization

## Abstract

**Background:**

The emergence of COVID‐19 has led to a significant public health crisis, and an increase in fungal infections, including candidemia. *Candida* species are frequently found in intensive care units (ICUs), and it is a common cause of death in many patients. The isolates were identified using polymerase chain reaction‐restriction. In this study, We investigated the factors linked to *Candida* infections in COVID‐19 burn patients in the ICU and assessed the antifungal susceptibility of the isolates in vitro.

**Methods:**

Out of 335 burn patients admitted to the ICU, fifty‐six with concurrent COVID‐19 were included in this study. A total of 133 yeast isolates were obtained from burn wounds, 29 from blood cultures, and 36 from urine cultures. The isolates were identified using polymerase chain reaction‐restriction fragment length polymorphism (PCR‐RFLP) analysis.

**Results:**

Out of fifty‐six patients, twenty‐nine had infections and forty‐eight had colonization, with *Candida parapsilosis* being the most common species. Twenty‐one patients died during their ICU stay, with mortality rates of 43.8% among colonized patients and 69.0% among infected patients. Fluconazole and itraconazole exhibited the highest minimum inhibitory concentrations, while luliconazole and amphotericin B were identified as the most effective antifungal agents.

**Conclusion:**

Our findings indicate that colonization may act as an important prognostic factor prior to the onset of candidemia. In addition, prolonged hospitalization, catheter use, and concurrent COVID‐19 infection were identified as key risk factors for candidemia in this patient group. Notably, the rising drug resistance in non‐*albicans Candida* species is a major public health concern.

## Graphical abstract

This study investigates the distribution of Candida species isolated from burn patients with candidemia, colonization, and urinary cultures. The findings highlight the predominance of *C. albicans* and *C. parapsilosis* across different sample types, including wound colonization, blood, and urine. Furthermore, species distribution was analyzed by body regions, providing valuable insights into the prevalence and anatomical patterns of fungal infections in burn patients. These results underscore the importance of targeted diagnostic and therapeutic approaches in managing fungal infections in this vulnerable population.

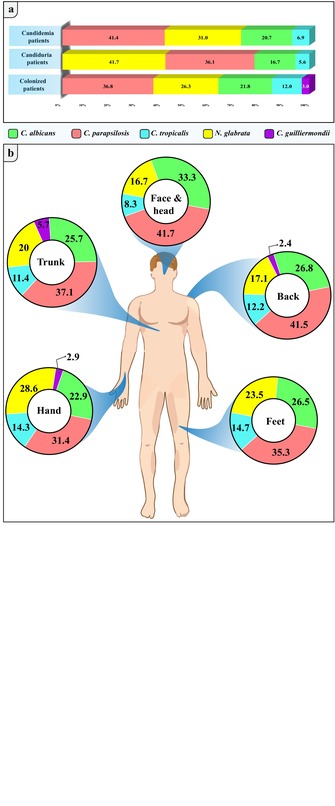

## 1 Introduction

The COVID‐19 pandemic has caused an unprecedented crisis in hospitals, particularly in ICUs, where patients are at high risk of developing fungal and bacterial co‐infections [1, 2]. The use of immunosuppressive drugs, such as anti‐interleukin‐6 agents and corticosteroids, in COVID‐19 patients can lead to *Candida* overgrowth, increasing the risk of candidemia‐related mortality [3, 4, 5, 6, 7]. This is because these drugs can cause the *Candida* species from the gastrointestinal tract to enter the bloodstream. In COVID‐19 patients, candidemia is associated with a high mortality rate [8]. Burn injuries compromise the protective skin and mucosal barriers, making patients vulnerable to severe bacterial (70%) and fungal (20%–25%) infections [9]. Candidemia is responsible for a significant proportion of deaths (30%–58%) in burn patients, making its management crucial. Several factors contribute to the high incidence of fungal infections in these patients. Factors such as a high *Candida* colonization rate, increased total body surface area (TBSA), and full thickness surface area (FTSA) in burn patients are linked to a higher incidence of fungal infections [10]. The leading cause of candidemia is *C. albicans* , while non‐*albicans Candida* species, such as *Candida parapsilosis, Candida (Nakaseomyces)* *glabrata* , and *C. krusei* , are becoming increasingly prevalent [11, 12]. Common risk factors for candidemia in both burn and COVID‐19 patients include the use of broad‐spectrum antibiotics, advanced age, total parenteral nutrition, central venous catheters, immunodeficiency, mechanical ventilation, chronic diseases such as diabetes, and prolonged hospitalization [1, 13, 14, 15, 16].

*Candida* species can develop resistance through various mechanisms, including point mutations in target genes, chromosomal alterations, and biofilm formation [12, 17]. There has been an increase in the occurrence of multidrug resistance among common *Candida* pathogens such as *C. albicans* , *N. glabrata, C. parapsilosis, C* *. tropicalis* , and *C. krusei* [7, 18, 19, 20, 21]. Additionally, resistance to fluconazole has been commonly documented during the COVID‐19 pandemic. Specifically, studies have shown that nearly half (48.4%) of candidemia infections exhibited resistance to fluconazole during the COVID‐19 outbreak [9, 22, 23].

According to the few studies that were conducted in the world and in Iran about fungal infections in patients with COVID, this study aims to investigate the prevalence of candidemia and *Candida* colonization along with the related risk factors in burn patients hospitalized in special care units in the three main burn centers in Iran.

## 2 Materials and Methods

### 2.1 Ethics Statement

The written informed consent was obtained from all participants who were able to provide it. For those unable to give consent themselves, permission was acquired from their legal representatives or guardians. Additionally, the study protocol received ethical approval from the Ethics Committee of Mazandaran University of Medical Sciences with approval number IR.MAZUMS.REC.1400.644.

### 2.2 Patients and Specimen Evaluation

A multicenter study was conducted from December 2021 to March 2023 at three major burn centers in Tehran, Rasht, and Sari. COVID‐19 diagnosis was confirmed through real‐time polymerase chain reaction (PCR) testing for SARS‐CoV‐2. Eligible participants were burn patients who had spent at least 2 weeks in the ICU and had second‐degree or higher burns affecting at least 10% of their TBSA. A comprehensive dataset was collected, including demographic details, medical history, antifungal treatment history, and clinical outcomes for further analysis. Patients who had incomplete data or failed to provide informed consent were excluded from the study.

### 2.3 Definitions

ICU‐acquired candidemia is diagnosed when at least one blood culture taken more than 2 weeks after ICU admission comes back positive for *Candida* species. Blood cultures are usually performed when there are signs of sepsis or suspected infection during clinical rounds [24].

Fungal wound colonization is identified when the same fungal species is isolated from at least three non‐sterile body sites, excluding different fungal species. The *Candida* colonization index (CI) measures the proportion of *Candida*‐colonized non‐sterile samples to the total number of cultured body sites. Patients are considered colonized if their CI is 0.5 or higher.

### 2.4 Sampling and Specimen Evaluation

Blood samples were incubated for 72 h in the BD BACTEC FX 40 automated Blood Culture System (Becton Dickinson Company, New Jersey, USA) to detect the presence of *Candida* species in patients with signs and symptoms of infection. If *Candida* species were found in at least one positive blood culture, it was considered *Candida* bloodstream infection. The positive blood culture bottles were cultured on Sabouraud Dextrose Agar (SDA; Laboratorios Conda Sa, Madrid, Spain) and CHROMagar *Candida* (CHROMagar, Paris, France), then incubated at 37°C for 24 to 48 h.

To detect colonization, urine and wound samples, including swabs and burn wound debris, were collected. The samples were then microscopically analyzed using 10% potassium hydroxide to identify yeast and mycelium. After that, the samples were cultured in SDA and CHROMagar at 37°C and monitored for 72 h. Finally, the colonies obtained from wound and blood samples were identified using microscopic morphology and Gram staining. *Candida* isolates were preserved at −80°C using trypticase soy broth containing 20% glycerol.

### 2.5 Molecular Identification

Total genomic DNA was extracted from fresh, pure fungal isolate cultures using the phenol‐chloroform glass‐beads method [23]. To identify the *Candida* species, polymerase chain reaction restriction fragment length polymorphism (PCR‐RFLP) method based on internal transcribed spacer (ITS) regions was used. The ITS regions were digested with the *Msp1* restriction enzyme [25].

### 2.6 In Vitro Antifungal Susceptibility Testing

Broth microdilution was used to test the susceptibility of various antifungal drugs against *Candida* isolates. We followed the Clinical and Laboratory Standards Institute (CLSI) guidelines M27‐A4 and M60. Thirteen antifungal drugs, such as itraconazole, fluconazole, miconazole, clotrimazole, voriconazole, posaconazole, amphotericin B, caspofungin, anidulafungin, nystatin, tioconazole, efinaconazole, and luliconazole, were evaluated for their in vitro activity.

Final concentrations of antifungal drugs ranged from 0.016 to 16 μg/mL for voriconazole, itraconazole, posaconazole, clotrimazole, miconazole, nystatin, tioconazole, efinaconazole, luliconazole, and amphotericin B. Concentrations ranged from 0.064 to 64 μg/mL for fluconazole and 0.008 to 8 μg/mL for caspofungin. To ensure quality control and reproducibility, tests included American Type Culture Collection control strains Pichia kudriavzevii (formerly *Candida krusei* ) *ATCC* 6258 and *C. parapsilosis* *ATCC* 22019. Minimum inhibitory concentrations were interpreted according to CLSI M27‐A4 and M60 breakpoints.

### 2.7 Statistical Analysis

The data were recorded using Microsoft Excel 2018 (Microsoft Corp, Redmond, WA, USA) and analyzed using SPSS software (version 22; SPSS Inc., Chicago, IL, USA). Quantitative variables were compared using the Mann–Whitney *U* test. Categorical variables were compared using Fisher's exact test to establish differences in distributions between the subgroups. A *p* value of ≤ 0.05 was considered statistically significant.

## 3 Results

### 3.1 Patient Population Characteristics

During the study period, 353 patients were admitted to the intensive care units (ICUs) of three burn hospitals. These patients were assessed for eligibility based on specific inclusion and exclusion criteria. From this group, 56 (15.9%) patients who contracted COVID‐19 during their hospital stay were selected for further investigation of risk factors and associated conditions. The male‐to‐female ratio was over 2.1, with men constituting 66.1% of the group. The most affected age group was 40–49 years. The detailed demographic and clinical characteristics of the patients are summarized in Table 1.

**TABLE 1:** Demographic characteristics and clinical factors associated with fungal involvement in 56 burn patients with COVID‐19 infection.

Characteristics	Involvement type [no. (%)]				
	Total patients (56) (100%)	Colonization	Candidemia*	No involvement	Statistical analysis
		[19 (33.9)]	[29 (51.8)]	[8 (14.3)]	
City					
Tehran	20 (35.7)	6 (31)	10 (34)	4 (50)	*p* = 0.686
Rasht	17 (30.4)	5 (26)	9 (31)	3 (37)	
Sari	19 (33.9)	8 (42)	10 (34)	1 (12)	
Gender					
Male	37 (66.1)	11 (57)	21 (72)	5 (62)	*p* = 0.638
Female	19 (33.9)	8 (42)	8 (27)	3 (37)	
Age categories					
0–9 years old	4 (7.1)	3 (15)	1 (3)	0 (0)	
10–19 years old	1 (1.8)	0 (0)	1 (3)	0 (0)	
20–29 years old	5 (8.9)	3 (15)	0 (0)	2 (25)	
30–39 years old	10 (17.9)	6 (31)	2 (6)	2 (25)	
40–49 years old	13 (23.2)	4 (21)	7 (24)	2 (25)	
50–59 years old	11 (19.6)	2 (10)	8 (27)	1 (12)	
60–69 years old	10 (17.9)	1 (5)	8 (27)	1 (12)	
70–79 years old	2 (3.6)	0 (0)	2 (6)	0 (0)	
Burn type					
Electricity burns	4 (7.1)	0 (0)	2 (6)	2 (25)	*p* < 0.0001*
Fire burns	14 (25.0)	5 (26)	6 (20)	3 (37)	
Explosion burns	22 (39.3)	10 (52)	9 (31)	3 (37)	
Water burns	11 (19.6)	2 (10)	9 (31)	0 (0)	
Chemical burns	5 (8.9)	2 (10)	3 (10)	0 (0)	
%TBSA range					
20–25	2 (3.6)	1 (5)	1 (3)	0 (0)	
30–35	16 (28.6)	9 (47)	3 (10)	4 (50)	
40–45	11 (19.6)	3 (15)	4 (13)	4 (50)	
50–55	11 (19.6)	3 (15)	8 (27)	0 (0)	
60–65	5 (8.9)	1 (5)	4 (13)	0 (0)	
70–75	4 (7.1)	0 (0)	4 (13)	0 (0)	
80–85	4 (7.1)	1 (5)	3 (10)	0 (0)	
90–95	3 (5.4)	1 (5)	2 (6)	0 (0)	
%FTSA					
II	10 (17.9)	4 (21)	5 (17)	1 (12)	*p* < 0.0001*
III	12 (21.4)	2 (10)	6 (20)	4 (50)	
II & III	32 (57.1)	13 (68)	16 (55)	3 (37)	
IV	2 (3.6)	0 (0)	2 (6)	0 (0)	
Candiduria					
No	20 (35.7)	6 (31)	8 (27)	6 (75)	
Yes	36 (64.3)	13 (68)	21 (72)	2 (25)	
*C. parapsilosis*	12 (21.4)	0 (0)	12 (41)	0 (0)	
*C. tropicalis*	2 (3.6)	0 (0)	2 (6)	0 (0)	
*N. glabrata*	10 (17.9)	0 (0)	10 (34)	0 (0)	
*C. guilliermondii*	0 (0.0)	0 (0)	0 (0)	0 (0)	
Candida Colonization					
*C. albicans*	9 (16.1)	4 (21)	5 (17)	0 (0)	
*C. parapsilosis*	17 (30.4)	6 (31)	11 (39)	0 (0)	
*C. tropicalis*	6 (10.7)	4 (21)	2 (7)	0 (0)	
*N. glabrata*	14 (25.0)	4 (21)	10 (35)	0 (0)	
*C. guilliermondii*	1 (1.8)	1 (5)	0 (0)	0 (0)	
Antibiotics Prophylaxis	56 (100.0)	19 (100)	29 (100)	8 (100)	
Diabetes	8 (14.3)	0 (0)	8 (27)	0 (0)	
Neutropenia	2 (3.6)	0 (0)	1 (3)	1 (12)	
Renal failure	2 (3.6)	2 (10)	0 (0)	0 (0)	
Skin graft	40 (71.4)	7 (36)	28 (96)	5 (62)	*p* < 0.0001*
Central venous catheters	39 (69.6)	11 (57)	27 (93)	1 (12)	*p* < 0.0001*
Receive nutrition type					
Oral	35 (62.5)	12 (63)	16 (55)	7 (87)	
ntravenous	21 (37.5)	7 (36)	13 (44)	1 (12)	
Alive	35 (62.5)	18 (94)	9 (31)	8 (100)	

*Note:* Total body surface area (%TBSA), Full thickness surface area (%FTSA). *p‐value of 5% or lower is considered statistically significant.

Burns caused by explosions (39.3%) were the most common type of burn injury. Notably, all patients received prophylactic antibiotics, corticosteroids, and mechanical ventilation. Of the 56 patients, 29 (51.8%) were diagnosed with candidemia, and 48 (85.7%) were found to have *Candida* colonization. Additionally, 46.5% (*n* = 27) of patients had a TBSA > 50%, and 57.2% (*n* = 32) had second‐ or third‐degree burns.

Figure S1 presents a comparison of different *Candida* species isolated from various types of burns in cases of candidemia, colonization, and candiduria. Explosion burns yielded the highest number of *Candida* species, while the lowest number was isolated from electrical burns. Notably, *Candida guilliermondii* was exclusively isolated from colonization cases.

### 3.2 Characteristics of Candidemia

**FIGURE 1:**
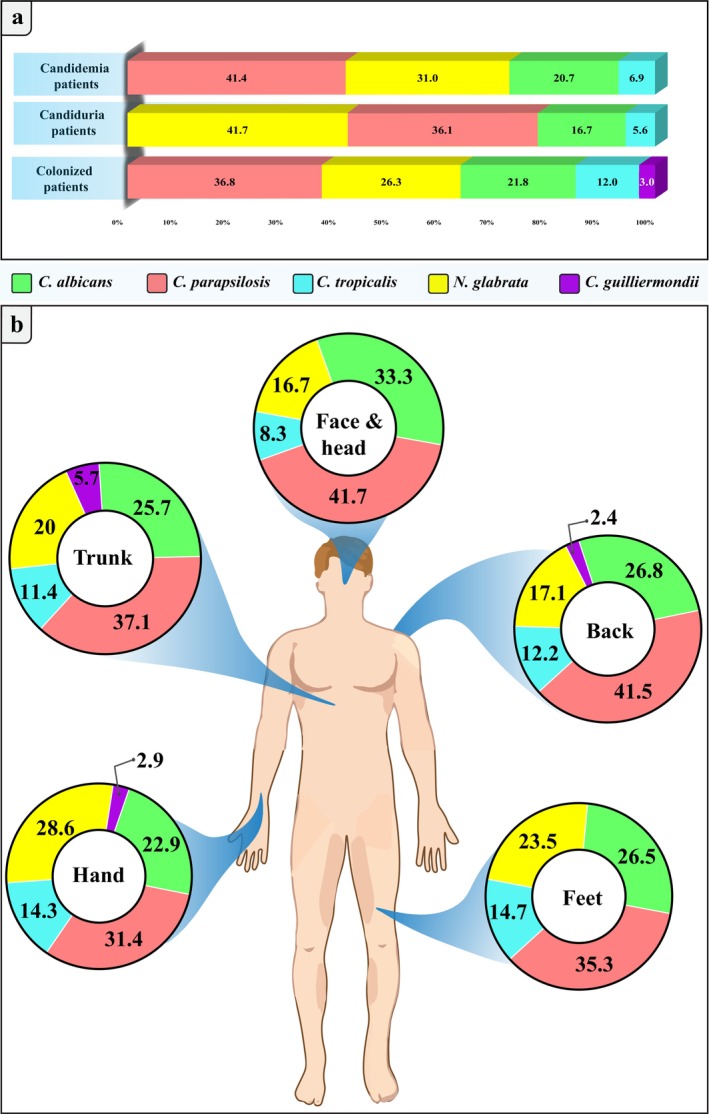
Distribution of *Candida* species isolated from burn patients in candidemia, colonization, urine culture. (a) Distribution of total *Candida* species isolated from wound colonization by number and percentage; (b) *Candida* species distribution across various body parts.

Out of the 56 COVID‐19 patients, 29 tested positive for candidemia through blood cultures. The male‐to‐female ratio was more than 2:1, with males constituting 72.4% of the patients. Patients diagnosed with candidemia were significantly older, with an average age of 50 ± 18 years, compared to other age groups (*p* < 0.0001). In this study, 96.5% of the patients underwent skin graft surgery. All patients required mechanical ventilation and received antibiotic prophylaxis as part of their treatment. Additionally, 93.1% of patients had an intravascular catheter, while 44.8% received total parenteral nutrition. Furthermore, TBSA was identified as a significant risk factor for candidemia (*p* < 0.0001). Notably, over 70% of the candidemia patients had TBSA involvement exceeding 50%, indicating a strong association between the extent of burns and the development of candidemia. The majority of cases were second‐ and third‐degree burns (48.3%). In the group of 29 patients diagnosed with candidemia, 29 distinct *Candida* isolates were identified as the causative agents of the infections. The most common species was *C. parapsilosis* with 12 isolates (41.4%), followed by *N. glabrata* with nine isolates (31%), and *C. albicans* with six isolates (20%). *C. tropicalis* was the least frequently isolated species, found in only two patients (6.9%). This distribution highlights the varying prevalence of different *Candida* species in candidemia cases. Figure 1a displays the distribution of *Candida* species isolated from blood cultures, burn wounds, and urine cultures.

### 3.3 Characteristics of Candida Colonization

In the course of this study, 133 isolates were collected from 48 patients, with *C. parapsilosis* and *N. glabrata* being the most frequently isolated species from wounds, accounting for 36.9% and 26.3% of the cases, respectively. Figure 1b illustrates the various *Candida* species isolated from different body sites.

Among the 56 COVID‐19 patients, 48 cases (85.8%) showed colonization. Of these, 29 patients (60.5%) had candidemia, and 34 patients (70.8%) had candiduria. The average hospital stay for colonized patients was 35 days (range: 21–63 days). The risk of colonization was higher in adults (43.75%) compared to other age groups. Additionally, 29 patients (60.0%) had second‐ and third‐degree burns, 35 patients (72.9%) had skin grafts, and 38 patients (79.2%) had a catheter.

### 3.4 Characteristics of Candiduria

Male patients accounted for twice the number of female patients (66.7%). The average age of these patients was 44 years, with the youngest being 3 years old and the oldest 78 years old. Among the 36 patients with positive urine cultures, 34 had positive wound colonization, and 21 had positive blood cultures.

A large proportion of patients (80.6%) required catheters, 13.9% had diabetes, and 72.2% underwent skin graft procedures. The average duration of hospitalization was 32 days, ranging from 21 to 63 days.

The most common species identified from urine cultures were *N. glabrata* (41.7%, *n* = 15) and *C. parapsilosis* (36.1%, *n* = 13). These two *Candida* species were prominently involved in urinary tract colonization and infections among the study population.

### 3.5 Antifungal Susceptibility

Out of 29 isolates, 37.9% showed resistance to fluconazole (geometric mean [GM] minimum inhibitory concentration [MIC] of 4.0 μg/mL), and 13.8% were resistant to voriconazole (GM MIC of 0.179 μg/mL). *C. albicans* and *C. parapsilosis* were the most commonly found fluconazole‐resistant species, with resistance rates of 50% (GM MIC of 6.35 μg/mL) and 25% (GM MIC of 3.175 μg/mL), respectively. Importantly, all isolates were susceptible to both caspofungin and anidulafungin. Table 2 provides a detailed summary of the antifungal susceptibility profiles for the different *Candida* species included in this study.

**TABLE 2:** The antifungal susceptibility of 29 *Candida* species isolated from burn patients with candidemia and COVID‐19 in Iran.

*Candida* species (*n*)	Antifungal agents	MIC parameters				
		Range	GM	MIC_50_	MIC_90_	Mode
Total (29)	AMB	0.016–1	0.135	0.125	0.5	0.125
	NYS	0.063–4	0.285	0.25	1	0.25
	FLC	0.25–64	4	4	32	2
	ITC	0.031–4	0.245	0.25	1	0.125
	MCZ	0.016–4	0.175	0.125	0.5	0.125
	Clo	0.016–4	0.135	0.125	0.5	0.063
	Tio	0.063–2	0.25	0.25	1	0.25
	VRC	0.016–4	0.179	0.125	1	0.063
	POS	0.016–0.5	0.129	0.125	0.5	0.125
	EFN	0.016–2	0.127	0.125	0.5	0.063
	Luli	0.016–0.5	0.062	0.063	0.125	0.063
	CAS	0.008–0.5	0.045	0.031	0.125	0.031
	Anid	0.008–0.25	0.039	0.031	0.125	0.031
*C. parapsilosis* (12)	AMB	0.063–0.5	0.149	0.125	0.25	0.125
	NYS	0.063–4	0.334	0.375	1	0.063
	FLC	0.5–64	3.175	4	8	2
	ITC	0.031–4	0.149	0.125	0.5	0.125
	MCZ	0.016–4	0.21	0.25	1	0.25
	Clo	0.016–1	0.149	0.125	0.5	0.5
	Tio	0.063–2	0.281	0.25	1	0.25
	VRC	0.063–4	0.158	0.125	0.5	0.125
	POS	0.016–0.5	0.118	0.125	0.5	0.125
	EFN	0.031–0.5	0.14	0.125	0.5	0.5
	Luli	0.016–0.25	0.047	0.125	0.125	0.031
	CAS	0.008–0.5	0.039	0.031	0.125	0.031
	Anid	0.008–0.25	0.032	0.125	0.063	0.063
*N. glabrata* (9)	AMB	0.031–0.5	0.17	0.125	0.5	0.125
	NYS	0.063–1	0.215	0.25	0.5	0.125
	FLC	0.‐64	6.35	8	16	8
	ITC	0.063–4	0.316	0.25	2	4
	MCZ	0.063–0.5	0.135	0.125	0.5	0.125
	Clo	0.016–1	0.092	0.125	0.5	0.125
	Tio	0.063–1	0.292	0.25	0.5	0.25
	VRC	0.063–4	0.368	0.5	1	1
	POS	0.016–0.5	0.079	0.063	0.5	0.063
	EFN	0.031–2	0.146	0.125	0.5	0.063
	Luli	0.016–0.5	0.063	0.063	0.125	0.063
	CAS	0.008–0.125	0.029	0.031	0.063	0.063
	Anid	0.008–0.125	0.043	0.031	0.125	0.031
*C. albicans* (6)	AMB	0.031–1	0.158	0.125	0.063	0.063
	NYS	0.125–2	0.707	0.5	2	1
	FLC	0.‐64	6.35	9	64	2
	ITC	0.063–4	0.446	0.5	2	1
	MCZ	0.063–1	0.281	0.125	1	0.125
	Clo	0.031–4	0.157	0.125	2	0.125
	TIO	0.063–1	0.281	0.25	1	0.25
	VRC	0.031–2	0.223	0.125	1	0.125
	POS	0.063–0.5	0.223	0.25	0.5	0.25
	EFN	0.016–0.5	0.063	0.078	0.25	0.25
	Luli	0.016–0.25	0.05	0.063	0.125	0.063
	CAS	0.008–0.5	0.04	0.063	0.5	0.063
	Anid	0.016–0.125	0.028	0.031	0.047	0.031
*C. tropicalis* (2)	AMB	0.016–0.5	0.045	0.0705	0.5	—
	NYS	0.125–4	0.177	0.1875	0.2375	—
	FLC	0.25–16	4	4	4	4
	ITC	0.063–1	0.354	0.5625	0.9125	—
	MCZ	0.031–0.5	0.089	0.094	0.5	—
	Clo	0.063–0.125	0.089	0.094	0.1188	—
	Tio	0.125–1	0.354	0.375	1	—
	VRC	0.063–0.25	0.177	0.1875	0.2375	—
	POS	0.031–0.5	0.25	0.3125	0.4625	—
	EFN	0.063–0.5	0.125	0.1565	0.5	—
	Luli	0.031–0.125	0.031	0.031	0.063	0.031
	CAS	0.016–0.031	0.031	0.031	0.031	0.031
	Anid	0.016–0.031	0.063	0.063	0.063	0.063

Abbreviations: AMB, Amphotericin B; CAS, Caspofungin; CLT, Clotrimazole; EFN, Efinaconazole; FLC, Fluconazole; ITC, Itraconazole; Luli, luliconazole; MCZ, Miconazole; NYT, Nystatin; POS, Posaconazole; VRC, Voriconazole.

Among the 133 isolates obtained from burn wounds of 56 COVID‐19 patients, 37.9% showed resistance to fluconazole with a GM MIC of 3.68 μg/mL, while 12% exhibited resistance to voriconazole with a GM MIC of 0.192 μg/mL. *C. parapsilosis* (19 cases) demonstrated the highest resistance to fluconazole with a GM MIC of 3.572 μg/mL, while *N. glabrata* (2 cases) was the most susceptible *Candida* species to fluconazole, with a GM MIC of 3.695 μg/mL. Table S1 provides a detailed description of these findings.

**FIGURE 2:**
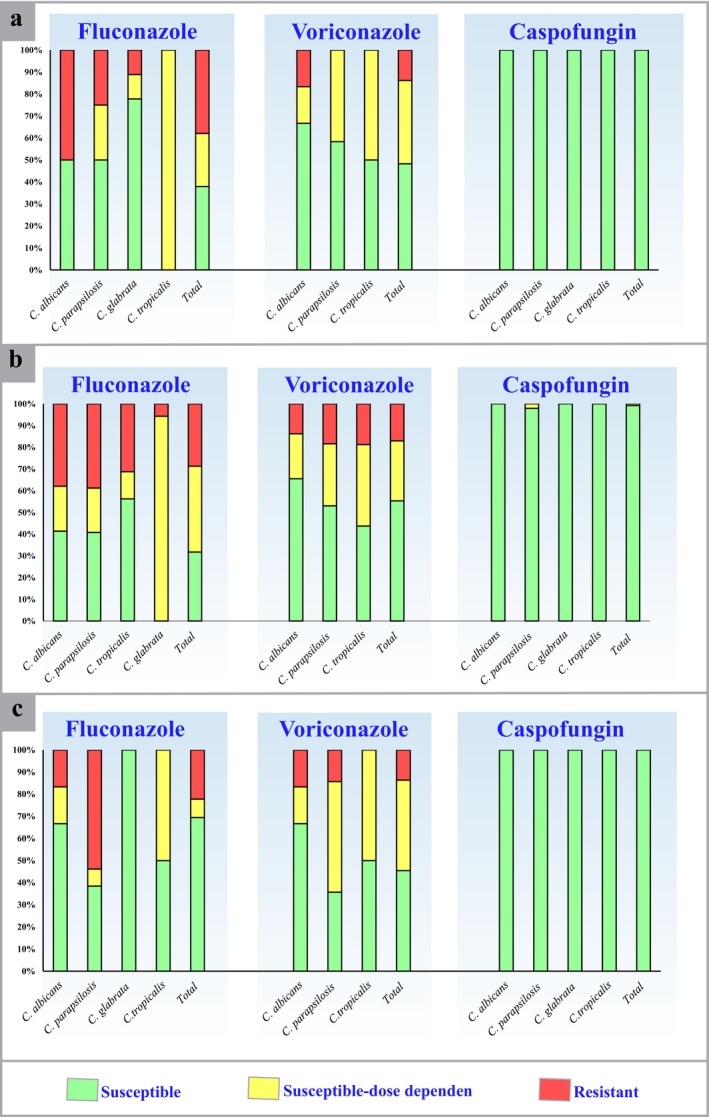
Antifungal drug resistance levels of *Candida* isolates obtained from wound (a) and blood (b) urine culture (c) samples of burn patients.

A total of 22.2% of the 36 urine culture isolates were found to be resistant to fluconazole, with a geometric mean (GM) minimum inhibitory concentration (MIC) of 2.776 μg/mL, while 8.3% were resistant to voriconazole, with a GM MIC of 0.227 μg/mL. Among the 13 isolates of *C. parapsilosis* , Seven isolates were identified as the most resistant *Candida* species, with a GM MIC of 4.694 μg/mL. Table S2 and Figure 2 provide a comprehensive overview of the antifungal susceptibility patterns observed in the different *Candida* species analyzed in this study.

### 3.6 Treatment and Outcome

Among the 56 burn patients with COVID‐19, 21 fatalities were recorded. Notably, 20 of these patients had *Candida* infections, and all of them exhibited *Candida* colonization. The mortality rates for patients with *Candida* colonization and infection were 43.8% and 69%, respectively. Moreover, 17 of the deceased patients were confirmed to have candiduria.

Of the 29 patients with candidemia, 65.5% (19 cases) were treated with caspofungin and amphotericin B, and 63.7% of them died.

The mean age of the deceased patients was 52 years, with the youngest being 7 years old and the oldest 78 years old. The 50–59 years age group experienced the highest mortality rate among all age groups. The average duration of stay in the intensive care unit (ICU) for the deceased patients was 16 days, ranging from 14 to 35 days. A significant proportion of the deceased patients had catheters (95.2%), skin grafts (90.5%), and diabetes (33.3%).

In this study, the highest mortality rates were associated with *N. glabrata* and *C. parapsilosis* , accounting for 28.6% (*n* = 24) and 25% (*n* = 21) of fatalities, respectively. These findings suggest a potential link between these specific *Candida* species and an increased risk of mortality in burn patients. Conversely, *C. albicans* exhibited a more favorable response to treatment compared to non‐*albicans Candida* species, highlighting the importance of accurate species identification and targeted antifungal therapy in managing candidiasis in burn patients.

Univariate analysis showed that the mortality rate had a significant relationship with gender, city, infection, and diabetes (*p* < 0.001).

## 4 Discussion

To the best of our knowledge, this is the first study globally to evaluate the prevalence of candidemia in burn patients with COVID‐19 hospitalized in the ICU. We identified several important findings in this study. All patients with candidemia (100%) had prior *Candida* colonization. Over 50% of COVID‐19 patients developed candidemia, and more than 80% exhibited *Candida* colonization. The geometric mean minimum inhibitory concentration (GM MIC) for fluconazole was notably high, while luliconazole was identified as the most effective antifungal, showing a lower GM MIC.

The study by Siegel et al. revealed that among 251 cancer patients with COVID‐19, a significant proportion (25.5%) of those in the elderly age group were also diagnosed with candidemia [26]. Similarly, in our current study, we observed that candidemia was more prevalent in the adult age group of COVID‐19 patients (23.3%).

In our study, the median time from ICU admission to the diagnosis of bloodstream infection was 17 days, with all infections occurring after at least 2 weeks, which aligns with the findings of Kaita et al. [27]. However, another study reported that the average time from burn injury to the onset of infection was either 7 days [28] or 8 days [29], which differs from our results. It is important to note that during hospitalization, the microorganisms causing the infection and their sensitivity to antimicrobial agents can change. Therefore, continuous monitoring of the microbiological status at each burn center is crucial [27].

In the present study, 64.3% of patients developed candiduria during their hospital stay. Notably, 72.5% of patients with candidemia also had concurrent candiduria. Furthermore, 48.3% of the species isolated from candidemia were identical to those found in cases of candiduria. However, it would be premature to conclude that they share a common portal of infection without further evidence.

In our study, the most common species isolated from candiduria were *C. albicans* (38.9%) and *C. parapsilosis* (27.8%), which aligns with findings from a study conducted in Turkey [30].

The present study found that patients with COVID‐19 often lacked common risk factors for candidemia, such as previous hospitalization, chronic liver diseases, and malignancy. However, prolonged hospitalization and the use of corticosteroids to manage inflammation and suppress the immune system contributed to an increased incidence of candidemia in these patients [31, 32].

Corticosteroids increase the binding of *Candida* species to epithelial cells, promote their proliferation in the intestines, and facilitate their migration from the gastrointestinal tract to the bloodstream [33, 34]. Recent studies in Turkey [3] and Brazil [33] have highlighted the significant role of corticosteroid use in the development of candidemia in COVID‐19 patients. Furthermore, research conducted in Japan [35] and the USA [36] has also reported an increase in candidemia associated with corticosteroid use. Severe COVID‐19 requiring hospitalization is likely a risk factor for candidemia [26].

In the current study, more than 70% of patients exhibited *Candida* colonization, and over 50% developed candidemia. It was observed that COVID‐19 patients who also developed candidemia had worse outcomes compared to those who only had COVID‐19. This difference in prognosis may be attributed to the use of immunosuppressive medications in the candidemia group, which could exacerbate the severity of COVID‐19 symptoms and complications [37]. Furthermore, all patients with candidemia were also colonized with *Candida* species. Our study observed a notable rise in candidemia cases among burn patients with COVID‐19 during the pandemic. Similar increases in candidemia cases have been reported globally among critically ill COVID‐19 patients [3, 38, 39, 40].

According to studies, the prevalence of *C. parapsilosis* has increased during the COVID‐19 pandemic [41, 42], which is consistent with our findings. In our study, *C. parapsilosis* was the predominant species isolated from cases of candidemia and colonization.

*Candida* colonization is a significant and independent risk factor for invasive *Candida* infection [43]. Skin colonization is the first step in invasive candidiasis [11, 44]. Moreover, the extensive use of antibiotics in burn patients contributes to increased fungal colonization in this population [45, 46]. *Candida* colonization is positive in half of intensive care unit patients and has a high predictive value for forecasting invasive infections [47]. In the present study, the most common *Candida* isolated from the wound cultures was *C. parapsilosis* . A key finding was that all infected patients were also colonized with *Candida*. Consistent with the findings of the present study, a study conducted in Iran reported a 69% prevalence of *Candida* colonization among COVID‐19 patients. This high prevalence suggests that COVID‐19 may be a significant risk factor for *Candida* colonization in hospitalized patients, potentially due to factors such as immune dysregulation, prolonged hospital stays, or the use of broad‐spectrum antibiotics and corticosteroids during treatment [48]. A study from Japan [27] found that 75% of the microorganisms identified in blood cultures were previously identified in swab cultures. This suggests that swab and wound culture results can help to predict the pathogens causing bloodstream infections in burn patients [27]. Additionally, Pittet et al. [43] demonstrated that patients with more than two sites of colonization have a risk of fungal infections, depending on the severity of colonization. Ostrosky‐Zeichner et al. [49] evaluated surgical patients in the ICU and found that patients with a *Candida* colonization index (CCI) above 0.4 who received prophylactic antifungal therapy had a significantly reduced risk of acquiring candidiasis [49]. Several studies have reported that primary *Candida* colonization is a significant risk factor for developing invasive candidiasis, and the risk increases with the number of colonized sites [50, 51, 52]. A study from Spain [53] reported 4 cases of candidemia among 88 simultaneous infections and superinfections. In another study from Italy [54], they reported 21 cases of candidemia among patients with COVID‐19. Several studies have shown an increase in candidemia in patients with COVID‐19 [53, 55, 56]. This increase may be due to various risk factors such as the use of corticosteroids and broad‐spectrum antibiotics, intravenous catheters, and dialysis [33]. COVID‐19 can disrupt the mucosal barrier, leading to the transfer of *Candida* species from the intestinal tract to the bloodstream, thus increasing the risk of candidemia [31]. According to the present study, the use of corticosteroids and broad‐spectrum antibiotics, indwelling catheters, prior *Candida* colonization, and mechanical ventilation were identified as the most important risk factors for developing candidemia, consistent with findings from other studies [55, 57].

There is growing concern about the occurrence of resistance in *Candida* species [20]. Reduced sensitivity to azoles results in the over‐prescription of echinocandins leading to increased resistance to echinocandins [20, 45, 58]. Studies have reported increasing antifungal drug resistance in *glabrata* and *C. auris* [57, 58, 59]. One study found that the resistance rates for strains isolated from candidemia were 22.9% for fluconazole and 8.6% for voriconazole. Additionally, a review of *Candida* isolates from around the world over a 20‐year period showed that *C. albicans* has the lowest frequency of antifungal drug resistance compared to other *Candida* species. However, our study revealed that the drug resistance rate of fluconazole in *Candida* species isolated from blood and colonization alone was 22.8% and 27.8%, respectively. Studies in Canada [22] and Spain [23, 42] reported resistance to fluconazole, which was consistent with our findings.

In the recent study, *C. parapsilosis* was found to be the most common *Candida* species, with a 36% resistance to fluconazole in cases of candidemia and colonization. A study in Spain observed a significant increase in the incidence of candidemia caused by fluconazole‐resistant *C. parapsilosis* compared to the pre‐COVID‐19 pandemic [42]. Another Spanish study reported an increase in *C. parapsilosis* resistance to voriconazole and fluconazole during the COVID‐19 period [24]. In the study conducted by Lazarescu et al. [45], it was noted that the majority of *Candida* strains in burn patients with candidemia showed resistance to fluconazole, itraconazole, and voriconazole.

The mortality rate in our patients was over 37.5%. Among patients with Candidemia consistent with other studies [26, 35, 36]. A study in Brazil reported a mortality rate of 73% in COVID‐19 patients with candidemia [35]. Across different continents, ICU mortality rates ranged from 59% to 75% and in‐hospital mortality rates. The mortality rate in our patients was over 37.5%. Among patients with infection, the mortality rate was 95.2%, consistent with other studies [26, 35, 36]. A study in Brazil reported a mortality rate of 73% in COVID‐19 patients with candidemia [35]. Across different continents, ICU mortality rates ranged from 59% to 75%, and in‐hospital mortality rates ranged from 50% to 69% [8, 60, 61].

Which was consistent with our study. Initiating appropriate antifungal therapy promptly is crucial in treating candidemia [62]. In our study, patients were treated with caspofungin and amphotericin B, and 37.5% of them passed away, likely due to COVID‐19 complications. Notably, our research revealed that when antifungal treatment was promptly administered, 17.3% of the patients with an infection were able to survive. This highlights the significance of early identification and prompt treatment of fungal infections in burn patients with COVID‐19. Burn injuries may result in immunosuppression and prolonged hospitalization, thereby increasing the risk of secondary infections, including candidemia [63]. COVID‐19 further exacerbates immunosuppression, complicating the clinical course of burn patients and making them more vulnerable to fungal infections [40]. Thus, considering the multiple risk factors already present in burn patients, COVID‐19 may serve as an important additional risk factor in these individuals.

Given that this study was conducted during the COVID‐19 pandemic, accessing patients proved challenging, and the sample size was consequently limited. Another limitation of the study was the absence of genetic analysis, which precluded a comparison of the relationship between *Candida* species isolated from blood and urine samples and skin wound colonization.

## 5 Conclusion

As far as we know, this is the first study to evaluate the prevalence of candidemia in burn patients with COVID‐19 admitted to the intensive care unit. This study highlights several critical factors related to candidemia in burn patients, which have important implications for clinical practice. Extended hospitalization, the use of catheters, the extent of TBSA involvement, and concurrent COVID‐19 infection were identified as key risk factors for the development of candidemia in this population. We also found that *Candida* colonization serves as a significant prognostic factor prior to the onset of candidemia. A concerning observation in our study was the increase in drug resistance and mortality rates among non‐albicans *Candida* species, which poses a major threat to public health.

## Ethics Statement

The study protocol was approved by the Institutional Research Ethics Committee of Mazandaran University of Medical Sciences (Approval no: IR.MAZUMS.REC.1400.644) along with the pertinent hospital research ethics boards.

## Conflicts of Interest

The authors declare no conflicts of interest.

## Supporting information


**Figure S1.** Distribution of total *Candida* species isolated from candidemia (a), colonization (b), urine culture (c) according to burn type.

**Table S1.** In vitro antifungal susceptibility of 133 fungal wound colonization strains isolated from 56 burn patients with COVID‐19 in Iran.
**Table S2.** In vitro antifungal susceptibility of 36 *Candida* strains isolated from urine cultures of burn patients with COVID‐19 in Iran.

## Data Availability

All data generated or analyzed during this study is included in the article.
