# Supplementary material for: Characterization of COVID‐19‐Associated Candidemia Among Burn Patients
Source: J Clin Lab Anal. 2025 Apr 8;39(10):e70031. doi: 10.1002/jcla.70031 (PMC12089798; doi:10.1002/jcla.70031)
Supplement: Supplementary file 1 — Figure S1. Distribution of total Candida species isolated from candidemia (a), colonization (b), urine culture (c) according to burn type. [file JCLA-39-e70031-s001.pdf]

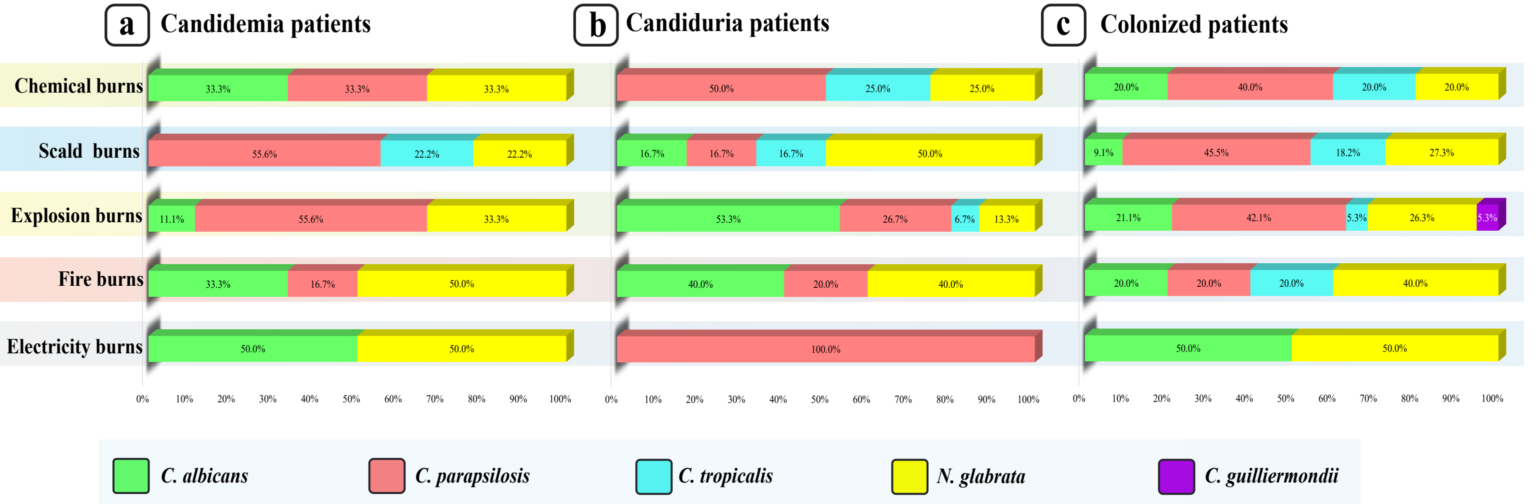

a Candidemia patients
b Candiduria patients
c Colonized patients
Chemical burns
Scald burns
Explosion burns
Fire burns
Electricity burns
33.3%
33.3%
33.3%
55.6%
22.2%
22.2%
11.1%
55.6%
33.3%
33.3%
16.7%
50.0%
50.0%
50.0%
50.0%
25.0%
25.0%
16.7%
16.7%
16.7%
50.0%
53.3%
26.7%
6.7%
13.3%
40.0%
20.0%
40.0%
100.0%
20.0%
40.0%
20.0%
20.0%
9.1%
45.5%
18.2%
27.3%
21.1%
42.1%
5.3%
26.3%
5.3%
20.0%
20.0%
20.0%
40.0%
50.0%
50.0%
0%
10%
20%
30%
40%
50%
60%
70%
80%
90%
100%
C. albicans
C. parapsilosis
C. tropicalis
N. glabrata
C. guilliermondii
