# Supplementary material for: Characterization of COVID‐19‐Associated Candidemia Among Burn Patients
Source: J Clin Lab Anal. 2025 Apr 8;39(10):e70031. doi: 10.1002/jcla.70031 (PMC12089798; doi:10.1002/jcla.70031)
Supplement: Supplementary file 2 — Table S1. In vitro antifungal susceptibility of 133 fungal wound colonization strains isolated from 56 burn patients with COVID‐19 in Iran. Table S2. In vitro antifungal susceptibility of 36 Candida strains isolated from urine cultures of burn patients with COVID‐19 in Iran. [file JCLA-39-e70031-s002.docx]

**Table S1.** *In vitro* antifungal susceptibility of 133 fungal wound colonization strains isolated from 56 burn patients with COVID-19 in Iran

| ***Candida* species(n)** | **Antifungal agents** | **MIC parameters** | | | |  |
| --- | --- | --- | --- | --- | --- | --- |
|  |  | **Range** | **MIC_50_** | **MIC_90_** | **GM** | **Mode** |
| Total isolates (n=133) | AMB | 0.016-1 | 0.125 | 0.25 | 0.120 | 0.125 |
|  | NYT | 0.063-4 | 0.25 | 1 | 0.344 | 0.25 |
|  | FLC | 0.25-64 | 4 | 16 | 3.680 | 8 |
|  | ITC | 0.016-8 | 0.125 | 1 | 0.203 | 0.125 |
|  | MCZ | 0.016-2 | 0.125 | 0.5 | 0.134 | 0.125 |
|  | CLT | 0.016-8 | 0.125 | 0.45 | 0.125 | 0.063 |
|  | Tio | 0.016-2 | 0.25 | 0.5 | 0.236 | 0.25 |
|  | VRC | 0.016-16 | 0.125 | 1 | 0.192 | 0.125 |
|  | POS | 0.016-4 | 0.125 | 0.5 | 0.141 | 0.125 |
|  | EFN | 0.016-2 | 0.125 | 0.5 | 0.116 | 0.25 |
|  | Luli | 0.016-0.25 | 0.063 | 0.125 | 0.058 | 0.063 |
|  | CAS | 0.008-0.5 | 0.031 | 0.125 | 0.035 | 0.031 |
|  | AFG | 0.008-0.25 | 0.031 | 0.063 | 0.033 | 0.031 |
| *C. parapsilosis* (n=49) | AMB | 0.016-1 | 0.125 | 0.25 | 0.120 | 0.125 |
|  | NYT | 0.063-4 | 0.25 | 1 | 0.346 | 0.25 |
|  | FLC | 0.25-32 | 4 | 16 | 3.572 | 8 |
|  | ITC | 0.016-8 | 0.125 | 0.5 | 0.217 | 0.125 |
|  | MCZ | 0.031-2 | 0.125 | 0.5 | 0.153 | 0.125 |
|  | CLT | 0.016-0.5 | 0.125 | 0.5 | 0.144 | 0.125 |
|  | Tio | 0.016-2 | 0.25 | 0.5 | 0.189 | 0.25 |
|  | VRC | 0.016-4 | 0.125 | 2 | 0.203 | 0.125 |
|  | POS | 0.016-4 | 0.125 | 0.5 | 0.149 | 0.125 |
|  | EFN | 0.016-1 | 0.125 | 0.5 | 0.151 | 0.25 |
|  | Luli | 0.016-0.25 | 0.063 | 0.125 | 0.060 | 0.063 |
|  | CAS | 0.008-0.5 | 0.031 | 0.125 | 0.033 | 0.031 |
|  | AFG | 0.008-0.125 | 0.031 | 0.063 | 0.034 | 0.031 |
| *C. tropicalis* (n=16) | AMB | 0.031-0.5 | 0.125 | 0.5 | 0.142 | 0.125 |
|  | NYT | 0.125-0.5 | 0.25 | 0.5 | 0.210 | 0.125 |
|  | FLC | 0.5-16 | 2 | 16 | 2.954 | 2 |
|  | ITC | 0.063-4 | 0.375 | 2 | 0.479 | 0.25 |
|  | MCZ | 0.063-0.5 | 0.1875 | 0.125 | 0.177 | 0.125 |
|  | CLT | 0.016-0.25 | 0.063 | 0.125 | 0.078 | 0.063 |
|  | Tio | 0.063-1 | 0.25 | 0.75 | 0.273 | 0.25 |
|  | VRC | 0.031-1 | 0.25 | 1 | 0.239 | 0.125 |
|  | POS | 0.031-0.25 | 0.125 | 0.25 | 0.105 | 0.125 |
|  | EFN | 0.016-2 | 0.25 | 0.5 | 0.211 | 0.25 |
|  | Luli | 0.016-0.125 | 0.031 | 0.063 | 0.030 | 0.016 |
|  | CAS | 0.008-0.125 | 0.031 | 0.125 | 0.037 | 0.031 |
|  | AFG | 0.016-0.063 | 0.031 | 0.063 | 0.030 | 0.031 |
| *C. glabrata* (n=35) | AMB | 0.031-0.5 | 0.125 | 0.5 | 0.133 | 0.063 |
|  | NYT | 0.063-4 | 0.25 | 1 | 0.350 | 0.25 |
|  | FLC | 0.25-64 | 4 | 16 | 3.695 | 4 |
|  | ITC | 0.031-4 | 0.125 | 1 | 0.147 | 0.063 |
|  | MCZ | 0.016-0.5 | 0.125 | 0.25 | 0.116 | 0.125 |
|  | CLT | 0.031-0.5 | 0.125 | 0.25 | 0.120 | 0.063 |
|  | Tio | 0.063-1 | 0.25 | 0.5 | 0.260 | 0.25 |
|  | VRC | 0.031-2 | 0.125 | 0.5 | 0.186 | 0.125 |
|  | POS | 0.016-1 | 0.125 | 0.5 | 0.120 | 0.125 |
|  | EFN | 0.016-2 | 0.125 | 1 | 0.123 | 0.25 |
|  | Luli | 0.016-0.25 | 0.063 | 0.25 | 0.072 | 0.063 |
|  | CAS | 0.008-0.125 | 0.031 | 0.063 | 0.030 | 0.031 |
|  | AFG | 0.008-0.25 | 0.031 | 0.125 | 0.033 | 0.063 |
| *C. guilliermondii* (n=4) | AMB | 0.063-0.125 | _ | _ | 0.075 | 0.063 |
|  | NYT | 0.25-0.5 | _ | _ | 0.297 | 0.25 |
|  | FLC | 16-32 | _ | _ | 22.627 | 32 |
|  | ITC | 0.5-2 | _ | _ | 1.000 | 1 |
|  | MCZ | 0.063-0.125 | _ | _ | 0.075 | 0.063 |
|  | CLT | 0.031-0.125 | _ | _ | 0.063 | 0.063 |
|  | Tio | 1-2 | _ | _ | 1.682 | 2 |
|  | VRC | 0.063-0.25 | _ | _ | 0.106 | 0.063 |
|  | POS | 0.063-0.125 | _ | _ | 0.089 | 0.063 |
|  | EFN | 0.016-0.31 | _ | _ | 0.026 | 0.031 |
|  | Luli | 0.063-0.063 | _ | _ | 0.063 | 0.063 |
|  | CAS | 0.016-0.063 | _ | _ | 0.031 | 0.031 |
|  | AFG | 0.008-0.016 | _ | _ | 0.011 | 0.016 |
| *C. albicans* (n=29) | AMB | 0.016-0.25 | 0.125 | 0.25 | 0.104 | 0.125 |
|  | NYT | 0.125-2 | 0.5 | 1 | 0.444 | 0.25 |
|  | FLC | 0.25-64 | 4 | 16 | 3.384 | 4 |
|  | ITC | 0.031-0.5 | 0.125 | 0.5 | 0.134 | 0.125 |
|  | MCZ | 0.031-2 | 0.125 | 0.6 | 0.119 | 0.125 |
|  | CLT | 0.031-8 | 0.125 | 1.2 | 0.145 | 0.063 |
|  | Tio | 0.031-1 | 0.25 | 0.5 | 0.217 | 0.25 |
|  | VRC | 0.031-16 | 0.125 | 1.2 | 0.175 | 0.125 |
|  | POS | 0.063-1 | 0.25 | 1 | 0.197 | 0.063 |
|  | EFN | 0.016-0.25 | 0.063 | 0.15 | 0.063 | 0.063 |
|  | Luli | 0.016-0.25 | 0.063 | 0.15 | 0.060 | 0.063 |
|  | CAS | 0.008-0.25 | 0.031 | 0.15 | 0.043 | 0.031 |
|  | AFG | 0.008-0.25 | 0.031 | 0.063 | 0.038 | 0.031 |

AMB: Amphotericin B, NYT: Nystatin, FLC: Fluconazole, ITC: Itraconazole, MCZ: Miconazole, CLT:Clotrimazole, VRC: Voriconazole, POS: Posaconazole, EFN: Efinaconazole, Luli: luliconazole, CAS: Caspofungin

**Table S2**. *In vitro* antifungal susceptibility of 36 *Candida* strains isolated from urine cultures of burn patients with COVID-19 in Iran

| ***Candida* species(n)** | **Antifungal agents** | **MIC parameters (µg/mL)** | | | | |
| --- | --- | --- | --- | --- | --- | --- |
|  |  | **MIC range** | **MIC_50_** | **MIC_90_** | **GM** | **Mode** |
| Total isolates (36) | AMB | 0.031-1 | 0.25 | 0.5 | 0.180 | 0.25 |
|  | NYT | 0.063-2 | 0.25 | 1.5 | 0.375 | 0.25 |
|  | FLC | 0.064-16 | 4 | 16 | 2.776 | 4 |
|  | ITC | 0.031-8 | 0.125 | 8 | 0.250 | 0.063 |
|  | MCZ | 0.031-2 | 0.125 | 0.25 | 0.149 | 0.25 |
|  | CLT | 0.031-4 | 0.125 | 0.5 | 0.128 | 0.125 |
|  | Tio | 0.063-2 | 0.25 | 0.5 | 0.286 | 0.25 |
|  | VRC | 0.031-2 | 0.25 | 1 | 0.227 | 0.25 |
|  | POS | 0.016-4 | 0.125 | 0.5 | 0.141 | 0.125 |
|  | EFN | 0.016-4 | 0.125 | 1 | 0.164 | 0.125 |
|  | Luli | 0.016-0.5 | 0.063 | 0.125 | 0.064 | 0.063 |
|  | Mil | 0.063-4 | 0.5 | 2 | 0.421 | 0.5 |
|  | Pep | 0.063-4 | 0.5 | 2 | 0.481 | 0.25 |
|  | CAS | 0.008-0.25 | 0.031 | 0.063 | 0.031 | 0.031 |
|  | AFG | 0.008-0.25 | 0.5 | 0.125 | 0.043 | 0.063 |
| *C. parapsilosis* (n=13) | AMB | 0.063-0.5 | 0.25 | 0.5 | 0.192 | 0.25 |
|  | NYT | 0.063-2 | 0.25 | 2 | 0.383 | 0.25 |
|  | FLC | 0.5-16 | 8 | 16 | 4.694 | 16 |
|  | ITC | 0.063-8 | 0.125 | 8 | 0.364 | 0.063 |
|  | MCZ | 0.031-1 | 0.125 | 0.25 | 0.155 | 0.25 |
|  | CLT | 0.031-1 | 0.125 | 0.5 | 0.155 | 0.063 |
|  | Tio | 0.063-0.5 | 0.25 | 0.5 | 0.250 | 0.25 |
|  | VRC | 0.063-2 | 0.25 | 0.5 | 0.225 | 0.25 |
|  | POS | 0.016-1 | 0.125 | 0.225 | 0.113 | 0.125 |
|  | EFN | 0.063-2 | 0.125 | 1 | 0.225 | 0.063 |
|  | Luli | 0.031-0.5 | 0.063 | 0.125 | 0.077 | 0.031 |
|  | Mil | 0.125-2 | 0.5 | 1 | 0.500 | 0.5 |
|  | Pep | 0.125-4 | 0.5 | 2 | 0.527 | 0.5 |
|  | CAS | 0.008-0.125 | 0.031 | 0.063 | 0.025 | 0.031 |
|  | AFG | 0.008-0.125 | 0.031 | 0.1126 | 0.037 | 0.031 |
| *C. glabrata* (n=15) | AMB | 0.031-1 | 0.25 | 0.5 | 0.218 | 0.5 |
|  | NYT | 0.063-2 | 0.25 | 1 | 0.315 | 0.25 |
|  | FLC | 0.064-16 | 4 | 8 | 2.301 | 4 |
|  | ITC | 0.031-8 | 0.125 | 1 | 0.208 | 0.125 |
|  | MCZ | 0.063-0.25 | 0.125 | 0.25 | 0.144 | 0.125 |
|  | CLT | 0.031-0.25 | 0.125 | 0.25 | 0.099 | 0.125 |
|  | Tio | 0.125-2 | 0.25 | 2 | 0.345 | 0.25 |
|  | VRC | 0.063-2 | 0.25 | 1 | 0.274 | 0.125 |
|  | POS | 0.031-0.5 | 0.125 | 0.4 | 0.125 | 0.125 |
|  | EFN | 0.016-4 | 0.25 | 2 | 0.229 | 0.125 |
|  | Luli | 0.016-0.5 | 0.063 | 0.5 | 0.069 | 0.063 |
|  | Mil | 0.125-4 | 0.25 | 2 | 0.435 | 0.25 |
|  | Pep | 0.125-4 | 0.5 | 1 | 0.500 | 0.25 |
|  | CAS | 0.008-0.063 | 0.032 | 0.063 | 0.029 | 0.031 |
|  | AFG | 0.016-0.25 | 0.063 | 0.125 | 0.050 | 0.063 |
| *C. albicans*(n=6) | AMB | 0.031-0.5 | - | - | 0.125 | 0.125 |
|  | NYT | 0.125-2 | - | - | 0.500 | 0.5 |
|  | FLC | 0.5-16 | - | - | 1.782 | 0.5 |
|  | ITC | 0.063-0.25 | - | - | 0.100 | 0.063 |
|  | MCZ | 0.031-2 | - | - | 0.177 | 0.063 |
|  | CLT | 0.031-4 | - | - | 0.158 | 0.063 |
|  | Tio | 0.063-0.5 | - | - | 0.199 | 0.25 |
|  | VRC | 0.031-2 | - | - | 0.177 | 0.125 |
|  | POS | 0.125-4 | - | - | 0.354 | 0.125 |
|  | EFN | 0.016-0.25 | - | - | 0.070 | 0.125 |
|  | Luli | 0.031-0.063 | - | - | 0.056 | 0.063 |
|  | Mil | 0.063-1 | - | - | 0.250 | 0.5 |
|  | Pep | 0.063-2 | - | - | 0.446 | 2 |
|  | CAS | 0.008-0.25 | - | - | 0.056 | 0.063 |
|  | AFG | 0.008-0.125 | - | - | 0.044 | 0.063 |
| *C. tropicalis* (n=2) | AMB | 0.031 | - | - | 0.089 | - |
|  | NYT | 0.063 | - | - | 0.500 | - |
|  | FLC | 0.064 | - | - | 1.414 | - |
|  | ITC | 0.031 | - | - | 1.414 | - |
|  | MCZ | 0.031 | - | - | 0.089 | - |
|  | CLT | 0.031 | - | - | 0.125 | - |
|  | Tio | 0.063 | - | - | 0.500 | - |
|  | VRC | 0.031 | - | - | 0.125 | - |
|  | POS | 0.016 | - | - | 0.089 | - |
|  | EFN | 0.016 | - | - | 0.022 | - |
|  | Luli | 0.016 | - | - | 0.016 | - |
|  | Mil | 0.063 | - | - | 0.500 | - |
|  | Pep | 0.063 | - | - | 0.250 | - |
|  | CAS | 0.008 | - | - | 0.044 | - |
|  | AFG | 0.008 | - | - | 0.031 | - |

AMB: Amphotericin B, NYT: Nystatin, FLC: Fluconazole, ITC: Itraconazole, MCZ: Miconazole, CLT:Clotrimazole, VRC: Voriconazole, POS: Posaconazole, EFN: Efinaconazole, Luli: luliconazole, CAS: Caspofungin
